# The Role of a Nutrition Support Team in the Management of Intestinal Failure Patients

**DOI:** 10.3390/nu12010172

**Published:** 2020-01-08

**Authors:** Lotte E. Vlug, Sjoerd C. J. Nagelkerke, Cora F. Jonkers-Schuitema, Edmond H. H. M. Rings, Merit M. Tabbers

**Affiliations:** 1 Erasmus Medical Center, Department of Pediatric Gastroenterology, Erasmus University Rotterdam, Sophia Children’s Hospital, 3015 GD Rotterdam, The Netherlands; 2 Amsterdam UMC, Department of Pediatric Gastroenterology, University of Amsterdam, Emma Children’s Hospital, Hepatology and Nutrition, 1105 AZ Amsterdam, The Netherlands; 3 Department of Pediatric Gastroenterology, Leiden University Medical Center, University of Leiden, Willem Alexander Children’s Hospital, 2300 RC Leiden, The Netherlands

**Keywords:** nutrition support team, intestinal failure, parenteral nutrition

## Abstract

Parenteral nutrition (PN) is a complex and specialized form of nutrition support that has revolutionized the care for both pediatric and adult patients with acute and chronic intestinal failure (IF). This has led to the development of multidisciplinary teams focused on the management of patients receiving PN: nutrition support teams (NSTs). In this review we aim to discuss the historical aspects of IF management and NST development, and the practice, composition, and effectiveness of multidisciplinary care by NSTs in patients with IF. We also discuss the experience of two IF centers as an example of contemporary NSTs at work. An NST usually consists of at least a physician, nurse, dietitian, and pharmacist. Multidisciplinary care by an NST leads to fewer complications including infection and electrolyte disturbances, and better survival for patients receiving short- and long-term PN. Furthermore, it leads to a decrease in inappropriate prescriptions of short-term PN leading to significant cost reduction. Complex care for patients receiving PN necessitates close collaboration between team members and NSTs from other centers to optimize safety and effectiveness of PN use.

## 1. Introduction

Intestinal failure (IF), first defined in 1981 as “a reduction in the functioning gut mass below the minimal amount necessary for adequate digestion and absorption of food” [1], is characterized by dependence on parenteral nutrition (PN). IF can be an acute self-limiting condition (type I), a prolonged acute condition requiring short-term PN for weeks or months (type II), or a chronic condition requiring long-term PN for months or years (type III) [2,3]. Chronic IF has different etiologies (see Table 1), but the most common cause in both adults and children is short bowel syndrome (SBS) as a consequence of extensive small bowel resection, followed by intestinal motility disorders and congenital enteropathies [4,5]. Specialized nutrition therapy is the corner stone of management of IF patients. The administration of macronutrients, micronutrients, fluids, and electrolytes can be given enterally via oral and tube feeding, and parenterally via a central venous catheter (CVC) or shunt. The enteral route is the optimal and most physiologic route of feeding and should always be extensively considered before PN is initiated. Feeding via the enteral route stimulates bowel adaptation and decreases the risk of IF-associated liver disease. If possible, patients should be fed orally since this prevents food aversion disorders, promotes epidermal growth factor release from the salivary glands, and increases gastrointestinal secretion of trophic factors [6]. Nutritional support is highly complex and several reports show that physicians are insufficiently trained and experienced to offer the necessary nutrition support [7,8,9]. This is also reflected in the current rudimentary role of nutrition education in medical training [10,11]. There are some clinical trials involving nutrition strategies in the pediatric and adult intensive care unit [12,13], but for patients receiving long-term PN, clinical practice is mostly based on experience and expert opinion [3]. Therefore, it is important that clinicians from different disciplines with expertise in IF collaborate in a nutrition support team (NST) to prevent complications and improve outcome. In this review we aim to discuss the historical aspects of IF management and NST development, and the role, composition and effectiveness of an NST in the management of adult and pediatric IF patients, with a focus on patients receiving long-term PN. Finally, we discuss the experience of our IF centers as an example of current practice in NSTs.

## 2. General Management of Intestinal Failure Patients

**Figure 1:**
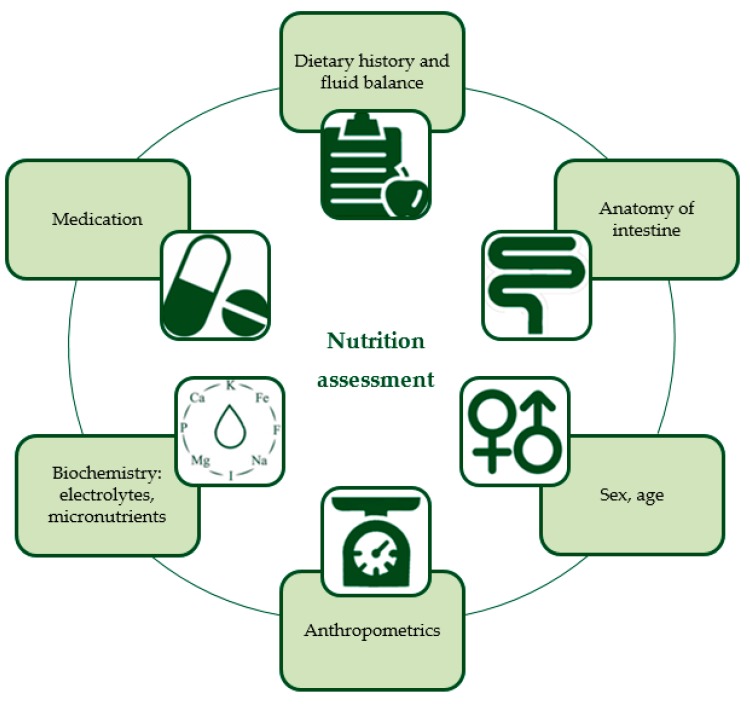
Nutrition assessment for intestinal failure patients on parenteral nutrition. This is a visual summary of the components described in more detail in Table 2.

At baseline, a comprehensive nutritional assessment is required to determine the hydration and nutritional status of a patient with IF and to calculate individual requirements. This nutrition assessment should incorporate aspects influencing intake, absorption, and expenditure, such as sex and age, the anatomy of the intestine (i.e., remaining bowel length, enterostomies, presence of ileocecal valve), and weight, height, and body composition. For visual representation see Figure 1, a more detailed description of the individual components is provided in Table 2 [14,15,16,17,18,19,20,21,22,23,24,25]. The assessment should be repeated regularly to evaluate the effectiveness of a nutrition intervention and to prevent patients from becoming dehydrated and undernourished with associated electrolyte abnormalities and weight loss. Especially in children, recurrent evaluation is essential to prevent growth deteriorations and to assess the adaptation response of the bowel and to increase enteral feeding and wean from PN to prevent excessive provision of calories [4,20].

The route, type, and duration of nutritional support depends on the patient, underlying disease, and stage of IF. In SBS, there are three clinical phases after surgery: the acute phase, adaptation phase, and maintenance phase, corresponding with different nutrition strategies [26,27].

The acute phase, directly following resection, is characterized by exclusive PN administration in a hospital setting. The intestine can recover and adapt, and hydration and nutritional status can be restored. In the adaptation phase, the ultimate goal of treatment is to increase enteral nutrition (EN) and wean from PN, since PN is associated with frequent complications such as IF- and PN-associated liver disease, central line-associated blood stream infections (CLABSIs), central line occlusions, central line-related thrombosis, metabolic bone disease, and fluid/electrolyte problems [28]. The maintenance phase reflects a period of stabilization. In some patients, enteral autonomy (i.e., being independent of PN) is achieved in hospital, others are discharged on home PN (HPN) when they are clinically stable. HPN can be indicated for patients with irreversible IF or for children who are expected to be dependent on PN for at least three months [27,29,30]. Patients with a longer residual small bowel, a younger age at intestinal resection, preservation of the ileocecal valve, necrotizing enterocolitis, absence of severe liver disease, and normal gastrointestinal motility have a greater chance of weaning off PN and achieving enteral autonomy, although it remains difficult to predict [31].

## 3. The Composition of a Nutrition Support Team

**Figure 2:**
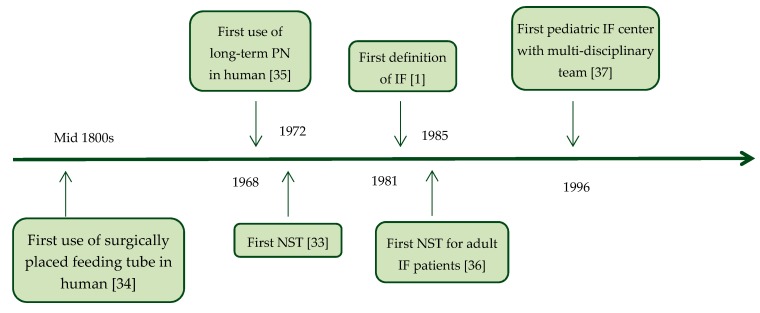
Timeline of “firsts” in the nutritional management of patients with intestinal failure [1,33,34,35,36,37]. IF: intestinal failure, NST: nutrition support team, PN: parenteral nutrition.

Each patient with IF that is malnourished, or at risk of becoming malnourished, will need artificial nutritional support in the form of EN and/or PN. The management of this nutritional support should be guided by an NST to improve quality of care [32]. The concept of an NST is not new. The first report of an NST was in 1972, set up to organize nutritional support better because of concerningly high sepsis rates in hospitalized patients on PN [33]. This was four years after the first use of PN in human, but before the first definition of IF (see Figure 2) [1,33,34,35,36,37]. In 1974, Butterworth described physician-induced malnutrition and the need to appreciate the role of nutrition in determining the outcome of illness for many patients in “The skeleton in the hospital closet.” This publication is nowadays seen as the cradle of nutrition teams [38]. An NST was described in 1992 in a report that led to the formation of the British Association for Parenteral and Enteral Nutrition (BAPEN) [39]: “A team of different disciplines with good communication enables nutritional support to be given in the best manner for each patient. Such a team improves the quality of treatment and reduces costs by: Avoiding unnecessary treatments and simplifying the treatments used; reducing complications; monitoring use of nutrients and outcome of treatment; reducing waste (for example, solutions prepared but not given or only partially used); standardizing nutrients and equipment to enable bulk purchase and negotiation of competitive rates”. A recently published review that thoroughly discusses the tasks, composition, and efficacy of NSTs in hospital for malnourished patients admitted to hospital shows favorable effects of nutritional support by NSTs [40].

In current society guidelines for both adults and children with IF, the management of HPN patients by an NST in centralized units is recommended by the ESPGHAN, ESPEN, ESPR, CSPEN, ASPEN, and AuSPEN [3,21,41,42,43,44,45]. In general, the structure and function of NSTs varies among institutions and depends on the local needs, available funding, organizational culture, and available personnel [46]. In specialized IF centers, the composition of the team also differs, as was shown in an international survey on pediatric IF teams. Of the 61 participating teams from 20 different countries the teams consisted of a pediatric gastroenterologist (present in 100% of the teams), dietitian (95%), nurse specialist (92%), pediatric surgeon (89%), pharmacist (82%), psychologist (66%), social worker (62%), speech therapist (48%), physiotherapist (38%), and general pediatrician (33%) [47]. For adults on HPN, the core members of an NST should be a physician, nutrition nurse specialist, senior dietitian, and senior clinical pharmacist, according to the ESPEN guidelines [43]. In Table 3 a suggestion for the composition of an NST for both pediatric and adult patients on HPN is given including the minimal designated roles of each team member [32,43,46,48,49]. The goal of an NST should be to provide both physical and psychological support for patients on HPN and their caregivers. The attention for psychological aspects is important, since HPN is known to adversely affect the quality of life and everyday problems such as the inability to work or to go to school or the inability to go on a holiday [50,51]. The tasks of the team are aimed at preventing and treating malnutrition, monitoring and evaluating nutrition therapy, and minimizing, managing, and auditing complications of EN and PN [43]. It should be emphasized that through interdisciplinary cross training, expertise, and experience of the suggested roles in Table 3 may vary between disciplines. For example, a clinical pharmacist might fulfill components of the roles of a dietitian, or a nurse may also have an expanded role including tasks of the supervising physician of the NST. Interdisciplinary cross training and the variation of tasks within the teams is essential to allow for safe practice in the absence of individual team members.

## 4. Improved Outcomes with Nutrition Support Teams

Several studies describe the effect of NSTs for patients suffering from IF on a variety of clinical outcomes which are summarized below. Studies were identified by performing an extensive search in PubMed in July 2019. Keywords included: intestinal failure, parenteral nutrition, multidisciplinary care (team), nutrition support (team), and intestinal rehabilitation program. Studies were included if they discussed the effects of a nutrition support team on patients receiving PN for IF. Furthermore, reference lists from relevant (review) articles were screened for studies missed in the primary search; relevant articles were included if they met the inclusion criteria.

### 4.1. Complications in Adults Receiving Parenteral Nutrition

As stated in Section 2, patients with IF are at risk of various complications, which can be related to the underlying intestinal disease, PN administration through a central venous catheter, or a combination of both, which can lead to significant morbidity and mortality [28]. The initiation of an NST has proven beneficial in the reduction of numerous complications in multiple studies as specified below.

As early as 1980, a decrease in complication rate was seen in a group of hospitalized adult patients receiving PN managed by an NST compared with a group managed by a variety of physicians [52].

Braun et al. retrospectively assessed the effect of initiation of an NST among hospitalized adult patients receiving PN. The NST consisted of a physician (gastrointestinal focused), two registered dietitian nutritionists, a registered nurse and a nutrition support pharmacist. Mortality decreased after the initiation of the NST (12.7 vs. 10.6%, *p* = 0.012). Furthermore, they reported fewer electrolyte disturbances after the implementation of the NST (53%; χ^2^ = 10.906, *p* = 0.004). Also, the authors reported on significantly less usage of potassium, phosphorus, and magnesium intravenous piggyback supplementation (88.8 vs. 94%; χ^2^ = 5.05, *p* = 0.026) [53].

A retrospective comparative study by Chong et al. on adult patients solely receiving PN in hospital showed a marked decrease in electrolyte disturbances after the initiation by the NST. The NST was led by a surgeon and included a surgical medical officer, a surgical ward pharmacist, a parenteral nutrition pharmacist, a dietician, and a nurse. Length of hospital stay, mortality, and duration of PN were similar prior and after initiation of the NST [54].

A systematic review by Naylor et al. published in 2004 included five studies (*n* = 489 patients) that reported on the effects of a total parenteral nutrition (TPN) team on metabolic and electrolyte abnormalities in adults receiving short-term PN in hospital [55]. Four studies (*n* = 420 patients) reported fewer abnormalities in patients managed by a TPN team (members of the teams varied between studies) [56,57,58,59]. One study (*n* = 69 patients) did not report a significant difference in the total number of abnormalities [60].

The positive effect of an NST on CLABSIs was observed in 78 adult patients who received PN on a surgical ward. NST consisted of a nutritional nurse (other members were not reported). Jacobs et al. describe a decrease from 24 to 3% in their cohort after initiation of an NST (*p* < 0.05) [59]. A study performed in a tertiary hospital in Michigan investigated patients receiving PN on wards where service of an NST was mandatory versus patients receiving PN on wards without an NST. CVC longevity was longer in units where NST consultation was mandatory (mean 20.4 days versus 14.4 days) [61].

Kennedy et al. also reported on the effect of an NST for adult patients receiving PN. After initiation of the NST mortality for patients receiving PN decreased from 43 to 24% (*p* = <0.05). Calculated cost savings was 50.715 British pounds in one year [62].

In conclusion, several retrospective studies report that in hospitalized adult patients receiving short-term PN an NST results in fewer electrolyte disturbances, complications not otherwise defined, CLABSI and mortality. These findings demonstrate that PN administration is not risk free and potentially lethal and should therefore be monitored by an NST. Since the cohorts of investigated patients differ between studies, and outcomes differ between studies, no meta-analysis of the results was possible.

### 4.2. Complications in Children Receiving Parenteral Nutrition

A Polish study published the effects of an NST on the prevalence of CLABSI in 27 children (age 2–48 months) receiving PN in hospital for a mean duration 219 days. 48% of the children suffered from a motility disorder, 37% had SBS. The study showed a decrease from 11.5 CLABSIs per 1000 catheter days prior NST to 1.1 CLABSIs per 1000 catheter days after NST initiation. Authors did not report on an effect on mortality [63].

Diamond et al. reported on the effect of a multidisciplinary team for children with SBS that included representation from surgery, neonatology, gastroenterology, transplantation, nursing, nutrition, pharmacy, social work, and palliative care. Children experienced significantly fewer overall episodes of sepsis per month of follow-up after implementation of the multidisciplinary team (0.3 vs. 0.5, *p* = 0.01). Other complications and mortality did not differ between the group before and after implementation of the multidisciplinary team [64].

The development of an advanced nutrition team for neonates with SBS (composed of professionals from gastroenterology, neonatology, general surgery, nursing, nutrition, pharmacy, social work, and occupational therapy) resulted in a decrease in mortality (14.8 versus 7.1%, *p* = 0.362) [65].

Modi et al. saw a similar effect on the survival in pediatric patients with severe SBS cared for by a multidisciplinary team compared to a historical control group with overall survival increasing from 70 to 89% (*p* < 0.05). No significant difference in weaning from PN was observed. The team consisted of dedicated pediatric staff in general surgery, gastroenterology, transplant surgery, nutrition, pharmacy, nursing, and social work [66].

Hess et al. also retrospectively identified the effect of a comprehensive clinical care team (pediatric surgeons, pediatric gastroenterologists, nutritionists, nurses, and pharmacologists) on mortality after 2 years of diagnosis in children with IF (underlying disease distribution not reported) (29.4 to 6.7%, *p* = 0.011) [67].

Oliveira et al. reported on reduced disease-related mortality after installation of a multidisciplinary intestinal rehabilitation program for children diagnosed with IF between 0–365 days with 0.6 deaths per 3 months (95% CI −1.23–0.02). No significant effect on CLABSIs or advanced liver disease was reported [68].

Multivariate analysis showed a hazard ratio of 5.6 for risk of death if an adult patient was cared for by an inexperienced HPN team compared to an experienced HPN team. In children a hazard ratio of 2.5 for risk of death was observed if a nutritional care team was absent during management [27].

A meta-analysis including 233 children performed by Stanger et al. in 2013 examining on the effect of multi-disciplinary rehabilitation programs for children with IF also found an increase in overall patient survival (RR = 1.22, 1.04–1.42, *p* = 0.005) [69].

In short, in pediatric patients receiving PN, an NST results in fewer CLABSIs, overall episodes of sepsis, and lower mortality across various studies. The increased survival rate as a consequence of an NST differs between studies but is high.

### 4.3. Prescription of Parenteral Nutrition

Several studies report on a reduction of inadequate use of PN after implementation of an NST. A reduction in the number of adult patients receiving short-term PN in both ICU and acute floor of a tertiary referral hospital and level 1 trauma center was observed by Parent et al. (RR, 0.64; 95% CI, 0.53–0.77 and RR, 0.80; 95% CI, 0.64–0.99, respectively). The rate of patients with short-duration PN use (PN duration of <5 days) declined by 30% in the ICU (RR, 0.70; 95% CI, 0.51–0.97). No data on mortality or clinical outcomes were reported [70].

Another study reported a reduction in TPN use in critically ill adult patients of up to 78.6% after implementation of an NST. Also less PN bags were wasted (493 versus 34) and yearly costs for PN were decreased from $513,246 to 195,176 [8].

Lee et al. reported that after initiation of an NST for adult patients receiving EN and/or PN for at least 72 h a higher percentage of goal energy (kcal) and protein (gram) provision was achieved (66.9 versus 86.2% and 67 versus 81.7% respectively). Goal energy and protein was calculated based on weight. Also, a decrease in days on PN and EN was observed. NST consisted of a physician, pharmacist, nurse, and dietician [71].

Piquet et al. reported that initiation of an NST in a university hospital resulted in a 35% decline in PN use with a parallel increase of EN. Furthermore, the amount of CLABSIs decreased from 25/year to 3/year (*p* = not reported). It was shown that the reduction in PN use and complications represented savings of 245.000 euro/year [72]. The observed decrease of PN usage is only seen in acute hospital setting and not observed when patients with chronic IF are treated by a multidisciplinary treatment team. A meta-analysis of three studies (*n* = 233 children [64,66,73]) in children with chronic IF due to SBS did not show an increase in the number of patients weaned from PN after implementation of a multidisciplinary pediatric IF team (RR = 1.05, 0.88–1.25, *p* = 0.62) [69].

In conclusion, an NST results in fewer short-term PN prescriptions for adult patients admitted to hospital resulting in significant cost saving. This effect is not described in children receiving long-term PN.

### 4.4. Discussion

It is shown that the initiation of an NST is associated with a decrease in mortality and in mechanical, septic, and metabolic complications for both pediatric and adult patients receiving either short- or long-term PN, and results in more appropriate PN prescription for adult patients receiving short-term PN. However, several methodological limitations of studies investigating the effect of NSTs should be taken into account. First, the majority of studies concerning NSTs are retrospective studies comparing a cohort of patients prior and post initiation of an NST. This imposes a significant risk of bias. Since two different time periods are compared, the observed effect cannot fully be attributed to the initiation of the NST. For instance, with improved catheter care and the advent of new lipid emulsions survival of patients has improved over time [28,74]. It should be noted that most improvements in care have been developed and researched by (members of) NSTs, however this review discusses the direct effect of an NST on patient care. Furthermore, several included studies have been published in the previous century. Therefore, the described effect of NSTs should be interpreted with caution. An idea for future research is to conduct multicenter prospective studies comparing the outcomes in patients with IF for different compositions of NSTs since there is a lot of variation in this composition between centers.

## 5. Experience of Two Dutch Intestinal Failure Nutrition Support Teams

The NSTs of the Amsterdam UMC Emma Children’s hospital and the Erasmus Medical Center Sophia Children’s hospital are embedded in an intestinal rehabilitation program (IRP). An IRP is an extended version of an NST for patients with IF which provides care with the goal to promote intestinal adaptation and to achieve enteral autonomy and oral feeding [75].

The multidisciplinary IF teams of Amsterdam and Rotterdam provide care for pediatric patients with chronic IF on HPN, and the team of Amsterdam also for adult patients with both acute and chronic IF. The teams consist of nurses, nurse specialists, a physician assistants, a pediatric gastroenterologist, a gastrointestinal surgeon, pediatric surgeons, an endocrinologist, dietitians, a pharmacist, a pediatric hematologist and a PhD student. Several other specialists, such as interventional radiologists, gastroenterologists for adults, speech therapists, social workers, psychologists and pediatric endocrinologists are consulted regularly. Approximately 115 adults suffering from type 3 IF and around 80 adults suffering from type 2 IF, and 65 children on HPN are managed by the teams. Also children and adults that have weaned off HPN are followed up by the teams.

For children, all patients/caregivers are trained in safely caring for the CVC and PN in hospital, prior to discharge with HPN. Adults are trained by homecare nurses. The IRP assesses the competence of the trained adult patient and determines whether homecare can be discontinued. Patients on HPN are seen at least twice a year at the outpatient clinic (in the first year after discharge more frequently). The outpatient consult is multidisciplinary, patients are seen by a physician, nurse, and/or a dietitian. In between outpatient consultations, patients are often seen in shared care by a general pediatrician, have phone consultations, give regular updates via email (for example the current weight of the patient) and can be seen earlier when indicated. Patients suffering from type 2 IF are seen every 6–8 weeks to determine optimal timing of reconstructive surgery.

The IF teams of both Amsterdam and Rotterdam are easily accessible, 24 h per day. Parents and adult patients can contact the team for PN delivery, feeding strategies, recipes or clinical assessment. The PN solutions are delivered by the hospital pharmacy (tailor made PN) or by a facility company (standard bags prepared or unprepared).

Monitoring of HPN patients involves medical and psychosocial evaluation. Annual screening includes physical examination, dietetic review, assessment of growth and body composition (with air-displacement plethysmography), laboratory monitoring (electrolytes, full blood count, kidney and liver function tests, coagulation profile, vitamin levels and trace elements), bone health (with hand X-ray and dual-energy X-ray absorptiometry (DXA), physical activity assessment), IF associated liver disease (with abdominal ultrasound and/or liver transient elastography (FibroScan)) and renal disease, dental review, endocrinological assessment, and, additional for pediatric patients, consultation of a speech therapist, physiotherapist, and social worker.

Twice a week, a multidisciplinary briefing takes place in which admitted patients and patients seen at the outpatient clinic are discussed. The pediatric IF team can be consulted by the clinicians from pediatric clinical wards if a patient is on PN for two weeks or more and not growing adequately. Adult patients from adult clinical wards including ICU are discussed on a weekly basis in the multidisciplinary nutrition team clinical round.

The close collaboration between the teams of Amsterdam and Rotterdam enables the discussion of difficult cases and collaboration on research topics. Also, the two remaining IF centers from The Netherlands, being Nijmegen and Groningen (the latter is the national intestinal transplantation center), are involved in this collaboration. Twice a year, the four teams come together to discuss clinical practice and ongoing studies. For optimization of care, members of both IF teams are involved in research projects, development of international guidelines, and attend national and international conferences. These conferences provide the teams the opportunity to present their study findings, to keep up to date on the management of IF, to work on team building, and to start and maintain collaborations in research with other IF centers from all over the world. Both teams are involved in the recently established European Reference Network for rare Inherited and Congenital Anomalies (ERNICA)—Intestinal Failure working group. Since IF is a rare disease, international collaboration is vital to improve care through research.

## 6. Conclusions

The care for both children and adults with IF is complex and requires expertise from different disciplines. Nutrition is the cornerstone of treatment in these patients that are dependent on short- or long-term PN. Clinicians should work together in an NST, consisting of at least a supervising physician (e.g., gastroenterologist, endocrinologist, and/or surgeon), nurse or nurse specialist, dietitian, and pharmacist. NSTs support the improvement of outcomes concerning survival, electrolyte disturbances, and infections. Also, NSTs lead to a decrease in the inappropriate prescription of short-term PN. Prospective multicenter studies are needed to improve the quantity and quality of evidence concerning outcomes with NSTs in IF patients. Close collaboration between team members and also between IF centers is crucial in the management of patients with this rare and complicated condition.

## Figures and Tables

**Table 1:** Common causes of intestinal failure in adults and children, adapted from [4,5].

Adults		Children	
Cause	Underlying Diseases	Cause	Underlying Diseases
Short bowel syndrome (extensive bowel resection)	Crohn’s disease Mesenteric infarction Radiation enteritis	Short bowel syndrome (extensive bowel resection or congenital)	Necrotizing enterocolitis Midgut volvulus Intestinal atresia Gastroschisis
Intestinal motility disorder	Chronic intestinal pseudo-obstruction syndrome (primary/secondary)	Intestinal motility disorder	Pediatric intestinal pseudo-obstruction syndrome Hirschsprung’s disease with involvement of small bowel
Congenital enteropathy	See underlying diseases in children	Congenital enteropathy	Microvillus atrophy Intestinal epithelial dysplasia Tricho-hepato-enteric syndrome Autoimmune enteropathy
Intestinal fistula	Iatrogenic Inflammatory Neoplastic Infectious Trauma		
Mechanical obstruction	Intraluminal Intrinsic bowel lesions Extrinsic lesions		

**Table 2:** Components of nutrition assessment for patients with intestinal failure.

Component	Description
Dietary history and fluid balance	Detailed information about previously tried diets including route, amount, and type of nutrition/formula with reasons for lack of success, and measurement of current fluid balance is required for designing a new individualized feeding regimen.
Anatomy of intestine	It is important to document the anatomy and function of the intestine or remaining intestine. Most nutrients are absorbed in the first part of the jejunum. In case of jejunum resection, the residual ileum is able to adapt and to partly take over the role of the jejunum in nutrient absorption. However, when the terminal ileum is resected, the reabsorption of vitamin B12 and bile salts cannot be replaced by jejunal cells. Resection of the ileocecal valve decreases intestinal transit time and supposedly predisposes to reflux of colonic content (including higher bacterial counts) back into the small intestine. Dysmotility and/or dilated loops cause intestinal stasis leading to SIBO, which negatively impacts the digestion and absorption of nutrients. [14]. High-output stomas may cause water, sodium, and magnesium depletion [15].
Energy requirements, anthropometrics, sex and age	Energy requirements are preferably measured by indirect calorimetry. If this is not possible, these requirements should be calculated based on body weight, height, sex, and age, and adjusted accordingly by patient response (i.e., when not gaining weight as expected). To measure the effect of a nutritional intervention, anthropometrics should be monitored with growth charts in pediatric patients. Next to weight and height, it is also recommended to assess and monitor body composition (with for example air-displacement plethysmography) and muscle function (with for example handgrip strength). In a recent study in pediatric IF patients receiving long-term PN, Neelis et al. reported that these children had higher fat mass and lower fat-free mass (i.e., muscle, water, bone, and internal organs), compared with healthy peers [16]. In another study, involving adult IF patients, it was shown that 73% had sarcopenia (i.e., loss of muscle mass and function) [17].
Biochemistry: electrolytes and micronutrients	Micronutrient deficiencies are common in IF patients [18,19]. Electrolytes such as sodium and magnesium may be low due to excessive gastrointestinal losses, whereas calcium, phosphate and potassium can be elevated as a consequence of dehydration [20]. Screening of electrolytes, vitamins, and trace elements should be performed at baseline and monitored thereafter. Electrolytes should be monitored every 1–3 months or more frequently when indicated (e.g., in the case of recent PN composition change or increased gastro-intestinal losses); vitamins and trace elements should be monitored every 6–12 months [21,22].
Medication	Some medication may increase intestinal losses (e.g., non-steroidal anti-inflammatory drugs, proton pump inhibitors, antibiotics) [23]. Proton pump inhibitors are frequently used to reduce gastric PH and gastric fluid production which is most markedly increased in the hypersecretory acute phase of IF [24]. Also, because of the decreased enteral absorption of nutrients and fluids by the small intestine, medication dosages may have to be adjusted or converted to intravenous supplementation. If it is uncertain whether the medication will be enterally absorbed, the intravenous route is the preferred one [25].

IF: intestinal failure, PN: parenteral nutrition, SIBO: small intestinal bacterial overgrowth.

**Table 3:** Suggested members of a nutrition support team for patients on home parenteral nutrition and their minimal designated roles [32,43,46,48,49].

**Core Members**	**Roles**
Supervising physician *	Supervision and overall responsibility of care provided by the team Understands underlying diseases and prognosis Prescribes PN solutions and medication
Gastroenterologist *	Understands and treats underlying diseases (Aids in) operative insertion of gastrostomies/jejunostomies
Surgeon *	Operative insertion of CVCs and gastrostomies/jejunostomies Surgical management of IF (e.g., restoration of bowel continuity, surgical lengthening procedures, and management of anastomotic strictures) Is responsible for postsurgical care
Interventional radiologist */Anesthesiologist *	Assists in challenging pediatric cases of central venous access. In adults, interventional radiologists are the primary consultant regarding CVC placement.
Nurse specialist *	Teaches and trains patients and/or caregivers in care of tubes, stomas, and CVCs and in home PN administration when applicable Recognizes and manages complications of CVCs etc. Is case manager for patients and their caregivers
Dietitian *	Conducts nutritional screening and assessment Designs and implements feeding regimens based on measurement or calculation of individual requirements Monitors patient’s response with nutritional, laboratory, and fluid status Is case manager for patients and their caregivers
Pharmacist *	Is responsible for providing enteral formulations and PN solutions and for composition optimization Advises on compatibility and stability issues and drug/nutrient interactions
**Additional Members**	**Roles**
Endocrinologist *	Advises on preventing and treating complications of PN (and malnutrition) such as growth problems in children, metabolic bone disease, osteoporosis, and diabetes mellitus
Hematologist *	Advises on prevention and treatment of catheter-related thrombosis
Psychologist *	Provides psychological support and therapy for patients (and caregivers)
Speech therapist *	Advises on oral feeding in case of oral aversion or swallowing difficulties
Social worker *	Provides emotional support for patients (and caregivers)
Physiotherapist *	Assesses motor development in pediatric patients Provides training programs focused on weight-bearing exercise

CVCs: central venous catheters, PN: parenteral nutrition. * If the nutrition support team cares for pediatric patients, this team member should be trained in pediatric medicine.
